# Diagnostic and prognostic value of ECG-predicted hypertension-mediated left ventricular hypertrophy using machine learning

**DOI:** 10.1097/HJH.0000000000004034

**Published:** 2025-05-23

**Authors:** Hafiz Naderi, Julia Ramírez, Stefan Van Duijvenboden, Esmeralda Ruiz Pujadas, Nay Aung, Lin Wang, Bishwas Chamling, Marcus Dörr, Marcello R.P. Markus, Choudhary Anwar A. Chahal, Karim Lekadir, Steffen E. Petersen, Patricia B. Munroe

**Affiliations:** a William Harvey Research Institute, Queen Mary University of London, Charterhouse Square; b Barts Heart Centre, St Bartholomew's Hospital, Barts Health NHS Trust, West Smithfield; c National Institute of Health and Care Research Barts Biomedical Research Centre, Queen Mary University of London, Charterhouse Square, London, UK; d Aragon Institute of Engineering Research, University of Zaragoza, Spain, CIBER-BBN; e Centro de Investigación Biomédica en Red - Biomateriales, Bioingeniería y Nanomedicina, University of Zaragoza, Spain; f Big Data Institute, La Ka Shing Centre for Health Information and Discovery, University of Oxford, UK; g Faculty of Mathematics and Computer Science, University of Barcelona, Spain; h School of Electronic Engineering and Computer Science, Queen Mary University of London, UK; i Department of Internal Medicine B, University Medicine Greifswald; j German Center for Cardiovascular Research (DZHK); k German Center for Diabetes Research (DZD) partner site Greifswald, Germany; l Center for Inherited Cardiovascular Diseases, WellSpan Health, Lancaster, Pennsylvania; m Department of Cardiovascular Diseases, Mayo Clinic, Rochester, Minnesota, USA; n Institució Catalana de Recerca i Estudis Avançats (ICREA), Barcelona, Spain

**Keywords:** electrocardiogram, hypertension, left ventricular hypertrophy, machine learning

## Abstract

**Objective:**

Four hypertension-mediated left ventricular hypertrophy (LVH) phenotypes have been reported using cardiac magnetic resonance (CMR): normal LV, LV remodelling, eccentric and concentric LVH, with varying prognostic implications. The electrocardiogram (ECG) is routinely used to detect LVH; however, its capacity to differentiate between LVH phenotypes is unknown. This study aimed to classify hypertension-mediated LVH from the ECG using machine learning and test for associations of ECG-predicted phenotypes with incident cardiovascular outcomes.

**Methods:**

ECG biomarkers were extracted from the 12-lead ECG of 20 439 hypertensive patients in UK Biobank (UKB). Classification models integrating ECG and clinical variables were built using logistic regression, support vector machine (SVM), and random forest. The models were trained in 80% of the participants, and the remaining 20% formed the test set. External validation was sought in 877 hypertensive participants from the Study of Health in Pomerania (SHIP). In the UKB test set, we tested for associations between ECG-predicted LVH phenotypes and incident major adverse cardiovascular events (MACE) and heart failure.

**Results:**

Among UKB participants 19 408 had normal LV, 758 LV remodelling, 181 eccentric and 92 concentric LVH. Classification performance of the three models was comparable in UKB. SVM (accuracy 0.79, sensitivity 0.59, specificity 0.87, AUC 0.69) was taken forward for external validation with similar results in SHIP. There was superior prediction of eccentric LVH in both cohorts. In the UKB test set, ECG-predicted eccentric LVH was associated with heart failure (hazard ratio 3.42, 95% CI 1.06–9.86).

**Conclusion:**

ECG-based ML classifiers represent a potentially accessible screening strategy for the early detection of hypertension-mediated LVH phenotypes.

## INTRODUCTION

Hypertension is the most common cause of left ventricular hypertrophy (LVH), and both are strong predictors of cardiovascular morbidity and mortality [1,2]. The diagnosis of hypertension-mediated LVH has relied on cardiac imaging, such as echocardiography and cardiac magnetic resonance (CMR) [3,4]. Using CMR imaging, four distinct hypertension-mediated LVH phenotypes have been reported: normal left ventricle (LV), LV remodeling, eccentric LVH and concentric LVH [5]. The spectrum of LVH phenotypes has been shown to have varying prognostic implications, with worse cardiovascular outcomes reported in eccentric and concentric LVH [6,7]. Due to the global burden of hypertension, a cost-effective approach in detecting LVH phenotypes is required to meet clinical demand. Before the advent of cardiovascular imaging, the electrocardiogram (ECG) had been used clinically to screen for LVH in hypertension [8–10]. However, its capacity to detect the four CMR-defined LVH phenotypes has not been explored.

Hypertension clinical guidelines recommend using the 12-lead ECG in individuals to screen for LVH [3,4,11]. The ECG is a readily available and low-cost first-line diagnostic tool performed on most patients during an acute care visit and follow-up of chronic cardiovascular conditions. In recent years, the transition to digitized ECG in electronic healthcare records has paved opportunities for ECG-based diagnostic and prognostic predictions. Moreover, the use of wearable technology and smartphones have increased its accessibility. Early detection of hypertension-mediated LVH can enable regular healthcare follow-up, rigorous cardiovascular risk management and timely initiation of effective blood pressure (BP)-reducing therapies. However, accurate reporting of the ECG is challenging for clinicians, and any improvement in automated analysis could ensure timely diagnosis and treatment of hypertensive patients with LVH [12–14]. A machine learning tool to detect hypertension-mediated LVH phenotypes could reduce the number of unnecessary CMR scans, allowing them to be used more efficiently, thus reducing waiting times. This is also of clinical significance as the ECG features derived from classifying hypertension-mediated LVH phenotypes may be used as surrogate markers to predict clinical outcomes in hypertensive patients.

In previous work, we have shown that supervised machine learning techniques can classify left ventricular hypertrophy derived from CMR with an area under the receiver operator curve (AUC) of 0.85 using support vector machine [15]. This study builds on that knowledge and explores the diagnostic and prognostic value of ECG-predicted hypertension-mediated LVH phenotypes using machine learning. We hypothesized that a selection of ECG biomarkers and clinical variables could classify hypertension-mediated LVH phenotypes defined by CMR imaging and that these ECG-predicted LVH phenotypes would be associated with incident cardiovascular outcomes.

## METHODS

### UK Biobank sample selection

The UK Biobank (UKB) is a large prospective population study where demographics, medication history, electronic health records, biomarkers, and genomics were collected in half a million participants aged 40–69 years when recruited between 2006 and 2010 from across the United Kingdom. The UKB imaging study was launched in 2015 with the aim of scanning 20% of the original cohort, that is 100 000 participants [16]. The UKB CMR protocol did not contain contrast administration, and details of the CMR protocol have been described previously [17]. The image analysis to derive volumetric data was performed using convolutional networks that has been detailed elsewhere [18].

**FIGURE 1:**
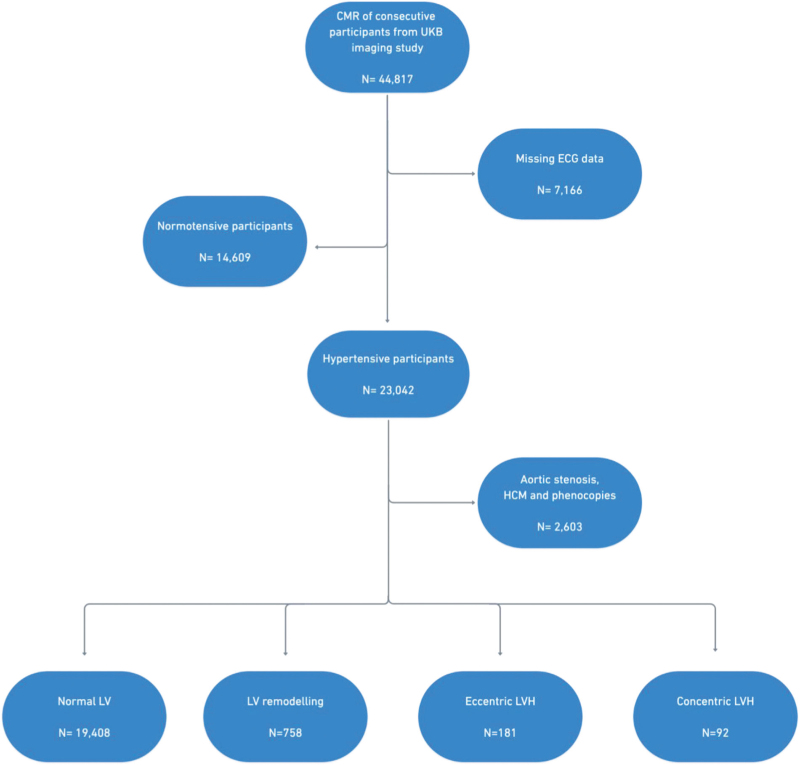
Flow diagram illustrating the steps involved in UK Biobank participant selection. CMR, cardiac magnetic resonance imaging; HCM, hypertrophic cardiomyopathy; LV, left ventricle; LVH, left ventricular hypertrophy.

A total of 44 817 participants had completed the UKB imaging study at the time of analysis. Of these, 37 651 participants had both ECG and CMR data available. Hypertensive participants (*N* = 23 042) were identified according to the ‘high normal’ BP grade of greater than or equal to130/85 mmHg based on the 2018 European Society of Cardiology/European Society of Hypertension (ESC/ESH) guideline [3]. The 130/85 mmHg BP cut-off was used instead of 140/90 mmHg as we were interested in identifying subclinical disease as the risk of hypertension-mediated LVH is on a continuous exposure scale. In UKB, BP readings from the imaging visit were analysed, as these were taken on the same day as the CMR study and 12-lead resting ECG. Each participant had two manual BP readings using a validated automated BP monitor or a manual sphygmomanometer. After calculating the average BP values, we adjusted for medication use by adding 15 and 10 mmHg to SBP and DBP, respectively, for participants reported to be taking BP-lowering medication [19,20]. We further defined hypertension by selecting relevant data fields from the UKB data showcase, including hypertension self-reported by participants, formal diagnosis from primary care physician and BP medication use. Participants with other causes of LVH (*N* = 2603) were excluded by reviewing exome sequence data for genes implicated in hypertrophic cardiomyopathy [21]. The remaining hypertensive participants (*N* = 20 439) were categorized into the four hypertension-mediated LVH phenotypes using mass-to-volume ratio. The CMR criteria used to define each hypertension-mediated LVH phenotype is shown in Table 1. Indexing for body surface area was performed using the Mosteller formula [22]. The data fields selected from UKB are shown in Supplementary Table 1. Figure 1 illustrates the UKB sample selection process.

**TABLE 1:** Definition of left ventricular phenotypes by cardiac magnetic resonance parameters

	Normal LV		LV remodelling		Eccentric LVH		Concentric LVH	
	Male	Female	Male	Female	Male	Female	Male	Female
Index LV mass (g/m^2^)	≤70	≤55	≤70	≤55	>70	>55	>70	>55
Indexed end diastolic volume (ml/m^2^)	≤110	≤94	≤110	≤94	>110	>94	≤110	≤94
LV mass : volume ratio (g/ml)	≤0.84	≤0.71	>0.84	>0.71	≤0.84	≤0.71	>0.84	>0.71

CMR, cardiac magnetic resonance imaging; LV, left ventricle; LVH, left ventricular hypertrophy. Indexing was performed using the Mostellar formula.

### ECG biomarker extraction

Full 15 s 12-lead ECG signals of each of the 20 739 participants were analysed semi-automatically using a custom algorithm written in MATLAB (version 2021a, Mathworks Inc., Natick, Massachusetts, USA) to derive 19 biomarkers (Supplementary Table 2) with a known physiological association with LVH, including conventional ECG-based LVH criteria, such as Sokolow–Lyon and Cornell criteria [23]. Only the independent ECG leads (I, II, V1–6) were analysed. Butterworth filter (1–45 Hz) was applied to attenuate baseline wander and high-frequency noise. ECG biomarkers from each independent lead were treated as individual features. In addition, global ECG features were calculated as the median value across the independent leads. The applied algorithm for extracting ECG biomarkers is detailed elsewhere [15].

### Ascertainment of clinical variables

In addition to ECG biomarkers, we also included clinical variables known to be associated with LVH (Table 2) in the classification model. Each clinical variable was defined by either a self-reported questionnaire or biochemistry results. Participants with serum total cholesterol of at least 5 mmol/l and haemoglobin A1c (HbA1c) at least 48 mmol/mol were considered to have hypercholesterolemia and diabetes mellitus, respectively. We corrected total and non-HDL cholesterol values for participants on cholesterol-lowering medication by dividing the total cholesterol by 0.73 and non-HDL cholesterol by 0.66 [24]. The presence of tobacco use was ascertained using self-reported questionnaires, with smoking status classified categorically as current, previous or never. Alcohol consumption was classified as current or never.

**TABLE 2:** Baseline characteristics of UK Biobank participants

	Overall (*N* = 20 439)	Normal LV (*N* = 19 408)	LV remodelling (*N* = 758)	Eccentric LVH (*N* = 181)	Concentric LVH (*N* = 92)	P-value
Age (years)	66 [11]	66 [11]	68 [10]	63 [13]	67 [9]	0.008
Sex (%)						**<0.001**
Female	9,335 (45.7)	8,744 (45.1)	457 (60.3)	77 (42.5)	57 (62.0)	
BMI (kg/m^2^)	26.7 [5.2]	26.6 [2.8]	28.5 [5.8]	25.9 [5.0]	27.3 [6.5]	**<0.001**
SBP (mmHg)	149 [24]	148 [23]	156 [26]	158 [27]	159 [30]	**<0.001**
DBP (mmHg)	85 [14]	85 [14]	88 [15]	86 [16]	90 [17]	**<0.001**
High cholesterol (%)	14,475 (70.8)	13,711 (70.6)	576 (76.0)	118 (65.2)	14,475 (76.1)	**0.03**
Total cholesterol (mmol/l)	5.0 [1.4]	5.0 [1.4]	5.0 [1.3]	5.0 [1.3]	5.0 [1.4]	0.9
Non-HDL cholesterol (mmol/l)	3.5 [1.3]	3.5 [1.3]	3.5 [1.2]	3.5 [1.3]	3.4 [1.3]	0.7
Diabetes (%)	1,422 (7.0)	1,277 (6.6)	121 (16.0)	12 (6.6)	12 (13.0)	**<0.001**
Smoking status (%)						**<0.001**
Never	12,059 (59.0)	11,518 (59.3)	392 (51.7)	99 (54.7)	50 (54.3)	
Previous	7,157 (35.0)	6,770 (34.9)	288 (38.0)	65 (35.9)	34 (37.0)	
Current	1,223 (6.0)	1,120 (5.8)	78 (10.3)	17 (9.4)	8 (8.7)	
Alcohol intake (%)						0.2
Never	908 (4.4)	852 (4.4)	46 (6.1)	7 (3.9)	<5 (3.3)	
Current	19 531 (95.6)	18 556 (95.6)	712 (93.9)	174 (96.1)	89 (96.7)	
Global ECG indices
Ventricular rate (beats/min)	63 [13]	62 [13]	70 [16]	56 [13]	63 [12]	**<0.001**
Sokolow–Lyon (%)	403 (2.0)	360 (1.9)	26 (3.4)	11 (6.1)	6 (6.5)	**<0.001**
Cornell voltage (%)	1,663 (8.1)	1,573 (8.1)	67 (8.8)	12 (6.6)	11 (12.0)	0.4
Pathological Q waves (%)	301 (1.5)	342 (1.8)	17 (2.2)	15 (8.3)	6 (6.5)	**<0.001**
ST segment deviation (mV)	0.01 [0.03]	0.01 [0.03]	0.02 [0.03]	0.01 [0.03]	0.01 [0.03]	0.8
QT dispersion (ms)	58 [48]	58 [47]	61 [58]	75 [44]	65 [56]	**<0.001**
Corrected QT duration (ms)	385 [31]	385 [31]	394 [28]	389 [36]	389 [30]	**<0.001**
P wave amplitude (mV)	0.05 [0.05]	0.05 [0.05]	0.04 [0.04]	0.06 [0.06]	0.05 [0.04]	**<0.001**
P wave terminal force in V1 (mV/ms)	−2.1[2.8]	−2.1[2.8]	−2.5 [2.9]	−1.7 [3.0]	−2.5 [3.5]	0.08
P wave duration (ms)	112 [22]	112 [22]	108 [20]	110 [28]	112 [15]	**0.01**
Q wave amplitude (mV)	−0.08 [0.3]	−0.08 [0.04]	−0.08 [0.05]	−0.09 [0.05]	−0.08 [0.05]	**0.02**
Q wave duration (ms)	23 [4]	23 [4]	23 [5]	25 [5]	24 [6]	**<0.001**
R wave amplitude (mV)	0.49 [0.23]	0.49 [0.23]	0.48 [0.22]	0.55 [0.31]	0.58 [0.31]	**<0.001**
S wave amplitude (mV)	−0.30 [0.19]	−0.30 [0.18]	−0.32 [0.21]	−0.42 [0.26]	−0.38 [0.26]	**<0.001**
QRS amplitude (mV)	0.92 [0.30]	0.92 [0.30]	0.92 [0.30]	1.15 [0.37]	1.14 [0.37]	**<0.001**
QRS duration (ms)	90 [16]	90 [16]	90 [18]	97 [16]	98 [15]	**<0.001**
QRS ascending slope (mV/s)	34.5 [15.1]	34.5 [15.4]	34.8 [15.3]	36.1 [18.3]	39.7 [17]	**<0.001**
QRS descending slope (mV/s)	−53.9 [−18.9]	−53.7 [18.5]	−55.5 [18.7]	−64.9 [22.0]	−66.6 [24.7]	**<0.001**
T wave amplitude (mV)	0.14 [0.07]	0.14 [0.07]	0.13 [0.06]	0.15 [0.08]	0.14 [0.08]	**<0.001**
T wave duration (ms)	108 [16]	108 [16]	108 [16]	110 [18]	109 [19]	**<0.001**

Counts variables are presented as number (percentage), continuous variables as median [interquartile range]. To assess for associations between participants with LVH-mediated LVH phenotypes, the Wilcoxon signed-rank test was used for continuous data and Fisher's exact test for categorical data. Global ECG indices are the median values calculated from the independent leads of the 12-lead ECG. Blood pressure and cholesterol values are adjusted for medication use. BP, blood pressure; LV, left ventricle; LVH, left ventricular hypertrophy. Statistically significant associations are in bold.

### Supervised machine learning techniques

Three supervised machine learning algorithms were evaluated for classification: logistic regression, support vector machine (SVM) and random forest. The algorithms were implemented in MATLAB, and the fit multiclass models for SVMs or other classifiers (fitcecoc) function was used to build the logistic regression and SVM classifiers [25]. The fit ensemble of learners for classification (fitcensemble) was used to build the RF classifier [26]. In our experiments, the dataset was randomly split into a training set (80%) for learning and a test set (20%) for performance evaluation. We applied 10-fold cross-validation to the training set. The metrics we used to assess classifier performance in the test set included: accuracy, sensitivity, specificity, precision, F1 score and AUC. The AUC in the multiclass setting was derived as weighted average of one vs. rest. The optimal threshold was chosen as the point that maximizes the model's differentiating ability when equal weight is given to sensitivity and specificity [27]. Further details of the models are described previously [15].

### External validation in Study of Health in Pomerania

SHIP is a population-based study investigating common risk factors and subclinical diseases from a random cluster sample drawn from the population of West Pomerania in Northeast of Germany [28,29]. SHIP consists of two independent cohorts: SHIP-START (recruited between 1997 and 2001) and SHIP-TREND (recruited between 2008 and 2012). Study participants for both cohorts were sampled from the general adult population aged 20–79 in West Pomerania. This study used the second follow-up of SHIP-START (SHIP-START-2) and baseline SHIP-TREND-0, as these were the studies with both 12-lead ECG and CMR data. The populations comprised 2333 participants for SHIP-START-2 and 4420 participants for SHIP-TREND-0 [29]. The CMR protocol used in the SHIP study has been described in a dedicated publication [30]. In brief, participants underwent contrast-enhanced CMR imaging, unless contraindicated in cases of allergy to contrast agent or impaired kidney function, and quantitative image analysis was performed using semiautomatic tools. A total of 1474 participants from SHIP had both CMR and ECG data. Blood pressure was measured three times in seated participants and the mean of the second and third reading was used. The same definition of hypertension was applied based on BP readings, medication use and diagnosis, yielding a total of 877 hypertensive participants in SHIP. The same ECG biomarkers and clinical features as per UKB analysis were extracted. For classification, the whole SHIP cohort was treated as a test set, therefore, down-sampling was not applied. The best-performing model (SVM) was taken forward for external validation in SHIP.

### Associations with cardiovascular outcomes in UK Biobank

Longitudinal data on clinical outcomes of UKB participants is recorded using linkage to Hospital Episode Statistics (HES) and the UK death register [31]. All UKB participants consented to be followed up. In this study, the primary endpoint was MACE defined as either hospitalization or death due to fatal/nonfatal myocardial infarction, stroke or ventricular arrhythmias. An additional analysis was performed testing for association with heart failure hospitalization separately. These clinical outcomes were selected due to their association with hypertension from the literature and clinical expertise. Cases were identified using relevant *International Classification of Disease, 9th or 10th Revision* (ICD-9, ICD-10), or *Office of Population Censuses and Surveys version 4* (OPCS 4) Classification of Interventions and Procedures codes in the health-related records or death register (Supplementary Table 3). The follow-up period was determined by the first appearance of ICD-9, ICD-10 or OPSC4 codes in either health record or death register data since the UKB imaging visit. Participants with prevalent events at the time of UKB enrolment were excluded from the survival analyses. Participants who did not experience an event were censored at death or the end of the follow-up period (30 November 2022).

### Statistical analyses

Statistical analyses were performed using R version 4.0.3 and RStudio Version 1.3.1093 [32]. After excluding missing or extreme ECG values (outside the range defined by the quartiles +/− 1.5 × interquartile range) the Classification And REgression Training (CARET) package in R was used for correlation analysis, and highly correlated ECG biomarkers were omitted (correlation coefficient threshold of +/− 0.9) [33]. ECG biomarkers with less than 10% of missing data were imputed using the Multivariate Imputation by Chained Equations (MICE) package in R [34]. The methodological support for the chosen threshold was based on previous published work [35,36]. There was an imbalance in the dataset, with the majority of participants having normal LV, and a minority having concentric LVH. In order to address the imbalance, down-sampling was applied using the CARET package in the training set to match the proportion of participants in the minority LVH group [33]. A chi-squared test was used to rank the features in terms of feature importance score.

Descriptive statistics are presented as median (interquartile range) for continuous variables or frequency (percentage) for categorical variables. The distribution of continuous data was assessed by visual inspection of the histograms and confirmed by the Shapiro–Wilk test. Baseline clinical and ECG characteristics of the hypertension-mediated LVH phenotypes were statistically compared with the normal LV group. To assess for associations, the ANOVA test was used for continuous data and the chi-squared test for categorical data. For all analyses, a two-tailed *P* value less than 0.05 was deemed statistically significant.

Associations between the ECG-predicted phenotypes and clinical outcomes were performed in the UKB test set (*N* = 3066) using multivariable-adjusted Cox proportional hazard regression, setting normal LV as the reference group. For each clinical outcome, the model was adjusted for age, sex and BMI, as these are the main covariates that influence LVH. Hazard ratios were reported with 95% confidence intervals (CI) to derive risk for each LVH phenotype compared to the normal LV group.

## RESULTS

### Study population

The clinical and ECG characteristics of the UKB participants stratified by hypertension-mediated LVH phenotypes are presented in Table 2. Among the 20 439 hypertensive participants in UKB, 19 408 (95.0%) had normal LV, 758 (3.7%) had LV remodelling, 181 (0.9%) eccentric LVH and 92 (0.5%) concentric LVH. Overall, the cohort had an average age of 66 years, and 46% were female. In the total hypertensive cohort, the frequency of participants with LVH criteria for Sokolow–Lyon and Cornell voltage on the ECG was 2 and 8%, respectively.

Table 3 shows the baseline characteristics of the Study of Health in Pomerania (SHIP) validation cohort. In SHIP, there were 877 participants with hypertension, of which 704 (80.3%) had normal LV, 134 (15.3%) LV remodelling, 12 (1.4%) participants with eccentric LVH and 27 (3.1%) with concentric LVH. The average age was 56 years, and 39% were female. Overall, the frequency of participants with LVH criteria for Sokolow–Lyon and Cornell voltage on the ECG was 6 and 9%, respectively.

**TABLE 3:** Baseline characteristics of particiants in the Study of Health in Pomerania

	Overall (*N* = 877)	Normal LV (*N* = 704)	LV remodelling (*N* = 134)	Eccentric LVH (*N* = 12)	Concentric LVH (*N* = 27)	*P* value
Age (years)	56 [19]	55 [20]	61 [16]	49 [27]	59 [15]	**0.02**
Sex (%)						**0.04**
Female	344 (39.2)	261 (37.1)	67 (50.0)	6 (50.0)	10 (37.0)	
BMI (kg/m^2^)	28.0 [5.4]	27.7 [5.4]	29.5 [5.1]	25.1 [4.3]	30.0 [5.8]	**0.007**
SBP (mmHg)	135 [18]	135 [18]	135 [22]	132 [22]	145 [20]	**<0.001**
DBP (mmHg)	82 [13]	82 [12]	81 [15]	75 [17]	83 [12]	0.3
High cholesterol (%)	693 (79.0)	542 (77.0)	121 (90.3)	8 (66.7)	22 (81.5)	**0.004**
Total cholesterol (mmol/l)	5.8 [1.4]	5.8 [1.3]	6.1 [1.4]	5.4 [1.6]	6.0 [1.5]	**0.01**
Non-HDL cholesterol (mmol/l)	4.4 [1.3]	4.3 [1.4]	4.6 [1.2]	4.0 [2.1]	4.7 [1.3]	**0.01**
Diabetes (%)	772 (88.0)	618 (87.8)	120 (89.6)	9 (75.0)	25 (92.6)	0.4
Smoking status (%)						**0.008**
Previous	728 (83.0)	597 (84.8)	105 (78.4)	9 (75.0)	17 (63.0)	
Current	149 (17.0)	107 (15.2)	29 (21.6)	<5 (25.0)	10 (37.0)	
Alcohol intake (%)						0.2
Never	53 (6.0)	41 (5.8)	10 (7.5)	<5 (16.7)	0 (0)	
Current	824 (94.0)	663 (94.2)	124 (92.5)	10 (83.3)	27 (100.0)	
[0,1-7]Global ECG indices
Ventricular rate (beats/min)	64 [14]	63 [14]	68 [13]	62 [10]	65 [16]	**0.04**
Sokolow–Lyon (%)	50 (5.7)	37 (5.3)	6 (4.5)	<5 (25.0)	<5 (14.8)	**0.004**
Cornell voltage (%)	75 (8.6)	62 (8.8)	10 (7.5)	<5 (8.3)	<5 (7.4)	0.9
ST segment deviation (mV)	0.03 [0.03]	0.03 [0.03]	0.03 [0.03]	0.03 [0.05]	0.03 [0.03]	0.7
QT dispersion (ms)	47 [45]	44 [45]	45 [51]	72 [22]	60 [39]	**0.04**
Corrected QT duration (ms)	384 [27]	384 [29]	387 [26]	378 [36]	387 [26]	0.2
P wave amplitude (mV)	0.02 [0.03]	0.02 [0.03]	0.02 [0.03]	0.01 [0.03]	0.03 [0.01]	0.7
P wave terminal force in V1 (mV/ms)	−2.8 [2.3]	−2.8 [2.3]	−3.2 [2.2]	−1.8 [1.6]	−2.5 [2.1]	0.5
P wave duration (ms)	86 [20]	86 [18]	86 [20]	79 [12]	86 [23]	0.8
Q wave amplitude (mV)	−0.10 [0.05]	−0.10 [0.05]	−0.09 [0.04]	−0.10 [0.06]	−0.12 [0.05]	0.3
Q wave duration (ms)	24 [4]	24 [4]	24 [4]	24 [3]	23 [4]	0.3
R wave amplitude (mV)	0.55 [0.24]	0.55 [0.23]	0.53 [0.26]	0.65 [0.18]	0.63 [0.38]	0.4
S wave amplitude (mV)	−0.24 [0.16]	−0.23 [0.16]	−0.25 [0.18]	−0.24 [0.13]	−0.28 [0.18]	0.1
QRS amplitude (mV)	0.98 [0.35]	0.98 [0.33]	0.96 [0.34]	1.22 [0.27]	1.20 [0.40]	0.7
QRS duration (ms)	90 [10]	90 [10]	89 [9]	90 [10]	92 [9]	0.1
QRS ascending slope (mV/s)	37.9 [14.9]	37.6 [14.3]	38.1 [17.3]	41.1 [9.0]	44.0 [25.9]	0.4
QRS descending slope (mV/s)	−55.1 [19.6]	−54.7 [18.8]	−54.8 [21.5]	−67.1 [21.9]	−62.0 [26.6]	0.1
T wave amplitude (mV)	0.16 [0.08]	0.16 [0.08]	0.15 [0.07]	0.16 [0.13]	0.14 [0.08]	0.6
T wave duration (ms)	112 [18]	112 [18]	108 [16]	120 [12]	110 [21]	0.4

Counts variables are presented as number (percentage), continuous variables as median [interquartile range]. To assess for associations between participants with LVH-mediated LVH phenotypes, the Wilcoxon signed-rank test was used for continuous data and Fisher's exact test for categorical data. Global ECG indices are the median values calculated from the independent leads of the 12-lead ECG. Blood pressure and cholesterol values are adjusted for medication use. BP, blood pressure; LV, left ventricle; LVH, left ventricular hypertrophy. Statistically significant associations are in bold.

### Machine learning model performance in UK Biobank

**FIGURE 2:**
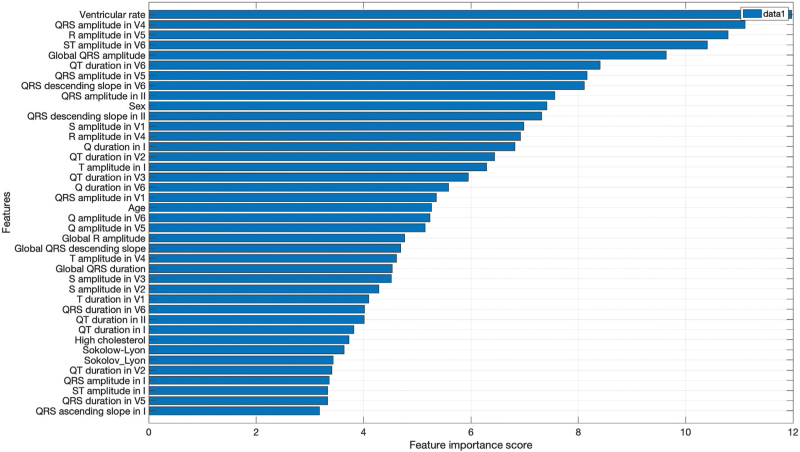
Ranking of the top 40 features using chi-squared feature selection.

Figure 2 shows the ranking of the top 40 features across all machine learning models using chi-squared feature selection. The top clinical features were sex and age and the highest ranking ECG predictors of LVH were ventricular rate and QRS amplitude in V4.

The performance metrics of all the supervised machine learning classifiers using both ECG and clinical variables in UKB are shown in Table 3. Classification with each method was comparable in the test set, with SVM showing 0.79 accuracy, 0.59 sensitivity, 0.87 specificity, 0.78 precision, 0.67 F1 score and AUC 0.69. Using SVM, the classification of eccentric LVH (AUC 0.86) and concentric LVH (0.72) was superior to normal LV (0.65) and LV remodelling (0.64) phenotypes, as shown in Fig. 2. The ECG biomarkers enhanced the model performance of the SVM classifier in UKB, compared to clinical variables alone (AUC values of 0.69 and 0.58, respectively), as illustrated in Fig. 2.

### External validation in Study of Health in Pomerania

**FIGURE 3:**
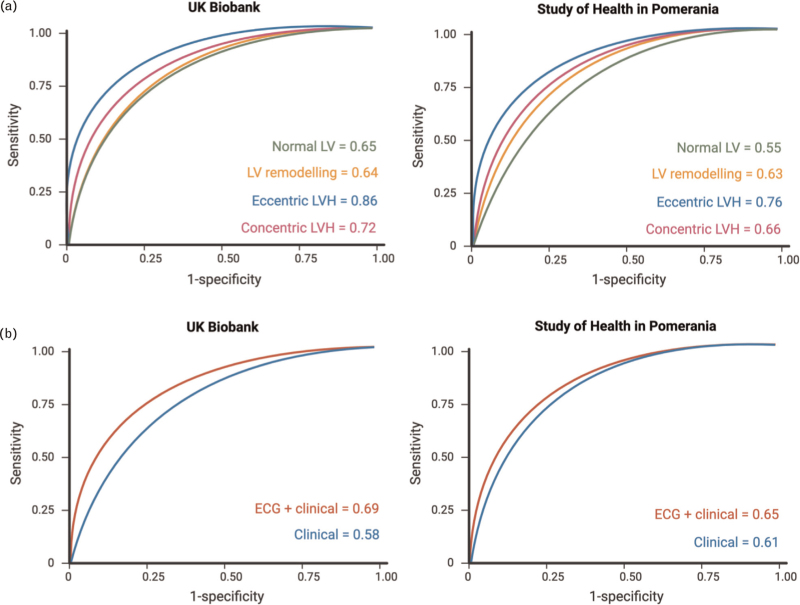
(a) Classification of hypertension mediated left ventricular hypertrophy phenotypes in UK Biobank and Study of Health in Pomerania using support vector machine. (b) Classification of hypertension mediated LVH phenotypes using ECG and clinical vs. clinical data alone in UK Biobank and Study of Health in Pomerania with support vector machine. ECG, electrocardiogram; LV, left ventricle; LVH, left ventricular hypertrophy.

External validation in the SHIP cohort using SVM showed a similar performance to UKB with 0.75 accuracy, 0.51 sensitivity, 0.85 specificity, 0.63 precision, 0.56 F1 score and 0.65 AUC (Table 4). Akin to UKB, the classification of eccentric LVH (AUC 0.76) and concentric LVH (0.66) was superior to normal LV (0.55) and LV remodelling (0.63) phenotypes. The ECG biomarkers also enhanced the model performance of the SVM classifier in the SHIP cohort, compared to clinical variables alone (AUC values of 0.65 and 0.61, respectively, shown in Fig. 3).

**TABLE 4:** Performance metrics of supervised machine learning classifiers using ECG and clinical variables in UK Biobank and validation testing in Study of Health in Pomerania

	UK Biobank			Validation in SHIP
	Logistic regression	Support vector machine	Random forest	Support vector machine
Accuracy	0.74	0.79	0.73	0.75
Sensitivity	0.47	0.59	0.45	0.51
Specificity	0.83	0.87	0.82	0.85
Precision	0.68	0.78	0.64	0.63
F1 score	0.56	0.67	0.53	0.56
AUC	0.71	0.69	0.70	0.65

AUC, area under the receiver operator curve; LVH, left ventricular hypertrophy; SHIP, Study of Health in Pomerania.

### Association of ECG-predicted left ventricular hypertrophy phenotypes with cardiovascular outcomes

**FIGURE 4:**
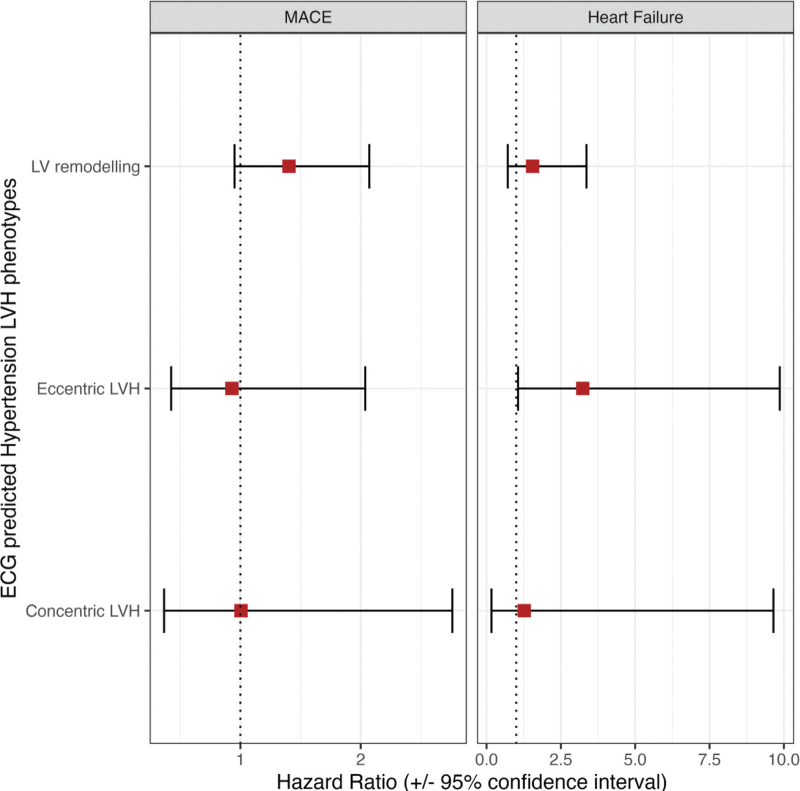
Associations of ECG-predicted hypertension-mediated left ventricular hypertrophy phenotypes and clinical outcomes. Results are hazard ratios from Cox hazards proportional regression models. The diseases listed are set as the model outcome (response variable) and hypertension-mediated LV phenotype in the exposure of interest with normal LV as the reference group. The model was adjusted for age, sex and BMI. CI, confidence interval; HR, hazard ratio; LVH, left ventricular hypertrophy; MACE, major adverse cardiovascular events.

Figure 4 shows the associations between ECG-predicted hypertension-mediated LVH phenotypes using SVM and the clinical outcomes of MACE and heart failure in the UKB test set (*N* = 3066). There was no statistically significant association with MACE (Supplemental Table 4); however, the hazard ratio of heart failure was 3.2 times higher (hazard ratio 3.24, 95% CI 1.06–9.86) in hypertensive patients with eccentric LVH with normal LV set as the reference group.

## DISCUSSION

In UKB, a combination of ECG biomarkers and clinical variables were able to discriminate between hypertension-mediated LVH phenotypes using supervised machine learning techniques. The machine learning classifiers had similar performances in UKB and external validation in the SHIP cohort using SVM demonstrated robustness of the model with reproducible results. We observed incremental value in using the 12-lead ECG compared to clinical variables alone for hypertension-mediated LVH detection. The classification of eccentric LVH and concentric LVH was superior to normal LV and LV remodelling phenotypes in both UKB and SHIP cohorts. Furthermore, we observed an association between ECG-predicted eccentric LVH group in the UKB test set and heart failure (hazard ratio 3.24, 95% CI 1.06–9.86), indicating potential clinical relevance of the model.

Ventricular rate and QRS amplitude in the precordial leads were the most influential ECG features in the model. Eccentric LVH had the lowest ventricular rate in both cohorts. Physiologically, this may be due to the dilated LV having reduced contractility and increased filling pressures [37,38]. The other top ECG predictors of LVH were measurements relating to the QRS complex, such as QRS duration, QRS descending slope and Sokolow–Lyon criteria, which are derived from amplitude measures of the QRS complex. Change in the QRS complex is a marker of electrical remodelling in LVH, which has been postulated to be due to the increase in the muscle mass of the LV mounting the forces of the LV potential. However, the increased QRS voltage is seen only in a minority of LVH cases in both clinical and animal studies, and consequently, voltage criteria have a high number of false-negative results and low sensitivity [39]. In prior work, ECG predictors of LVH have suffered low sensitivity, ranging from 15 to 30% [40]. Using a combination of ECG and clinical variables, our SVM model sensitivity values were over 50%, without compromising specificity (87%).

Previous efforts have classified LVH by ECG using deep learning approaches with comparable results [41–43]. However, this is the first study to classify hypertension-mediated LVH using supervised machine learning, and there is no direct comparative study. Beneyto *et al*. (2023) also compared supervised machine learning approaches in detecting hypertension as the cause of LVH, and found SVM to be superior to decision tree and random forest models [44]. The authors defined LVH as maximal LV wall thickness greater than 12 mm in diastole and found that SVM had the optimal balance between specificity of 86% and sensitivity of 31%. Beneyto *et al.* used a combination of clinical, laboratory and echocardiographic variables in their models. They identified SBP and the number of antihypertensive medications among the most significant features for classification. Although ECG features were not included in the study, their findings offer a methodological comparison relevant to our research, particularly in terms of balancing model sensitivity and specificity.

We validated our findings in an independent cohort, and the results demonstrated robustness of the machine learning model. Although both cohorts were European, they had differing clinical profiles. Compared to UKB, the SHIP cohort was younger (66 vs. 56 years) and had a higher incidence of hypercholesterolemia (19 vs. 79%). The SHIP cohort is noted to be a ‘high-risk’ population compared to the relatively ‘healthy’ cohort of UKB. Despite the differing risk profiles of these cohorts, our model's comparative performance indicated there is potential translatability to community populations. Nevertheless, the prevalence of LVH in both these cohorts was relatively low indicating the need for further validation experiments in populations with more cases of LVH.

Although echocardiography is the more accessible imaging modality for assessing LVH, it is operator-dependent, and poor acoustic windows can limit its use [45,46]. CMR is considered the gold standard in the assessment of LVH as it is accurate, reproducible and noninvasive [47,48]. We used CMR for this study as this was available in both UK Biobank and SHIP. The CMR protocols in UK Biobank and SHIP had distinct protocols with slight differences in downstream image analysis [49,30]. For example, in UKB, the papillary muscles were excluded from the LV mass measurement in contrast to SHIP. These differing approaches may explain the relatively low prevalence of LVH in the hypertensive population of UKB (5%) compared to SHIP (20%) considering the mean SBP in UKB was 149 mmHg compared to 135 mmHg in SHIP. This may also explain the slight drop in model performance using SVM in UKB (AUC 0.69) and SHIP (AUC 0.65), as the model was trained in UKB potentially introducing an element of overfitting. Despite these differences, we speculate that the superior prediction of concentric LVH and eccentric LVH in both cohorts is due to the distinct geometry of these phenotypes on imaging. A dilated LV characterizes eccentric LVH, while concentric LVH is synonymous with a thickened LV wall and small LV cavity size. These findings will need further support with validation in other datatsets.

Following the validation of the model in the SHIP dataset, we were interested in assessing whether the ECG-predicted phenotypes were associated with clinical outcomes. Using the test set in UKB (*N* = 3066), we observed a significant association between eccentric LVH and heart failure with a 3.2 increased hazard rate of heart failure compared to those with normal LV geometry. Hypertension leads to heart failure through LVH and LV diastolic dysfunction, eventually progressing to LV systolic impairment in a subset of patients in the presence of chronic volume and pressure overload [50,51]. Nauta *et al.*[38] have shown that patients with heart failure with eccentric LVH have a clinical and biomarker phenotype that is distinctly different from those with concentric LVH. In a retrospective post hoc analysis of 1015 patients with heart failure (LV ejection fraction <40%), the majority of patients (*N* = 873) had eccentric LVH and were, on average, younger (*P* = 0.005) and had a lower ejection fraction (*P* < 0.001). The authors also found that beta-blocker up-titration was associated with a mortality benefit in heart failure with eccentric but not concentric LVH (*P* < 0.001). In our study, we also found that participants with eccentric LVH were on average younger in both cohorts. The lack of association with outcomes in the other LVH phenotypes is likely due to the relatively short follow-up period in UKB and population size of the test set. However, with the UKB aiming to scan 100 000 participants, there is potential to review outcomes in the future with greater numbers.

Although the results from this analysis are promising, further development and testing are required before clinical implementation. For clinical applicability, the model's predictive value would need to be optimized, if successful machine learning methods could be integrated into a point-of-care application or directly into ECG machines. Using such automated approaches may then be used for population screening to enhance clinician ECG interpretation and expedite workflow by ensuring that advanced imaging tests are used for those who need it most, thereby reducing unnecessary testing and subsequent waiting times. Developing a model for single lead ECG would also be of interest, particularly in the era of wearable and smartphone technology. Hypertension guidelines recommend the 12-lead ECG is performed in all patients newly diagnosed with hypertension [52]. This provides an opportunity to compare the cost–benefit of ECG-based machine learning classification of LVH to that of conventional management. Important challenges will be to compare different sensitivity and specificity thresholds for the model to balance the trade-off between diagnostic accuracy and the economic benefit of downstream testing with potential false-positive results. In order to implement an ECG-based machine learning screening strategy for LVH, it will be important to evaluate the cost-effectiveness under various clinical and cost scenarios.

An important limitation is that both cohorts are predominantly white European ancestry, therefore, further work is warranted to elucidate the classification of hypertension-mediated LVH in other ethnicities. Furthermore, although we were able to perform external validation of the SVM model, there is a need to externally validate the association with cardiovascular outcomes. Our experiments included only ECG and clinical characteristics as features in the machine learning models. The rationale for this was to incorporate features that are accessible in a wide range of healthcare settings. Nevertheless, there is potential to include additional features to improve model performance and further personalize the machine learning algorithm. The UKB has access to numerous biomarkers and healthcare data such as BP medication history, biochemistry results, metabolomics, and genetic data, including genetic risk scores. These data could be incorporated into models for further development. In this study, we used three supervised machine learning approaches, and these models can be developed further to increase accuracy. Models using XGBoost (Extreme Gradient Boosting), decision trees and K-nearest neighbor are attractive options [53–55]. Agnostic approaches, such as unsupervised machine learning and deep learning, may also be used to identify novel signals in the ECG associated with LVH.

In conclusion, ECG-based classifiers could discriminate between the four hypertension-mediated LVH phenotypes with external validation demonstrating robustness. We also observed an association between the ECG-predicted eccentric LVH and heart failure indicating there is potential prognostic information gained from the model. If predictions can be further improved, this methodology would enhance the capabilities of nonspecialists and potentially represents an accessible screening strategy for the early detection of hypertensive patients with LVH.

## ACKNOWLEDGEMENTS

This study was conducted using the UK Biobank resource under access application 2964. We would like to thank all the participants, staff involved with planning, collection and analysis, including core lab analysis of the CMR imaging data. Figure 3 was created using Biorender.com.

Sources of funding: H.N. was supported by the British Heart Foundation Pat Merriman Clinical Research Training Fellowship (FS/20/22/34640). J.R. acknowledges fellowship RYC2021-031413-I from the European Union ‘NextGenerationEU/PRTR’ and MCIN/AEI/10.13039/501100011033. N.A. acknowledges support from Medical Research Council for his Clinician Scientist Fellowship (MR/X020924/1). S.E.P. acknowledges the British Heart Foundation for funding the manual analysis to create a cardiovascular magnetic resonance imaging reference standard for the UK Biobank imaging resource in 5000 CMR scans (www.bhf.org.uk;PG/14/89/31194). S.E.P. and P.B.M. acknowledge support from the National Institute for Health and Care Research (NIHR) Biomedical Research Centre at Barts (NIHR202330). S.E.P., K.L. and E.R. have received funding from the European Union's Horizon 2020 research and innovation programme under grant agreement No 825903 (euCanSHare project). K.L. has received funding from the European Union's Horizon 2020 research and innovation programme under grant agreements No 101080430 (AI4HF project) and No. 101057849 (DataTools4Heart project). The Study of Health in Pomerania (SHIP) is part of the Community Medicine Research net (CMR) (http://www.medizin.uni-greifswald.de/icm) of the University Medicine Greifswald, which is supported by the German Federal Ministry of Education and Research (BMBF, grant number: 01ZZ96030 and 01ZZ0701) and the Federal State of Mecklenburg-West Pomerania. MRI scans in SHIP-2 and SHIP-TREND-0 have been supported by a joint grant from Siemens Healthineers, Erlangen, Germany and the Federal State of Mecklenburg-West Pomerania. This study was carried out in collaboration with the German Centre for Cardiovascular Research (DZHK), which is supported by the German Federal Ministry of Education and Research (BMBF).

### Ethics statement

This study complies with the Declaration of Helsinki; the work was covered by the ethical approval for UK Biobank studies from the NHS National Research Ethics Service on 17th June 2011 (Ref 11/NW/0382) and extended on 18 June 2021 (Ref 21/NW/0157) with written informed consent obtained from all participants. The work related to Study of Health in Pomerania is via application reference number SHIP/2023/31/D. The study is covered by the overall ethical approval for SHIP studies approved by the Ethics Committee at the University Medicine Greifswald, Germany.

### Conflicts of interest

S.E.P. provides consultancy to and owns stock of Cardiovascular Imaging Inc, Calgary, Alberta, Canada.

## Supplementary Material

Supplemental Digital Content

## Data Availability

The data underlying this article were provided by the UK Biobank under access application 2964. UK Biobank will make the data available to bona fide researchers for all types of health-related research that is in the public interest, without preferential or exclusive access for any persons. All researchers will be subject to the same application process and approval criteria as specified by UK Biobank. For more details on the access procedure, see the UK Biobank website: http://www.ukbiobank.ac.uk/register-apply/.

## References

[1] Aronow WS. Hypertension and left ventricular hypertrophy. *Ann Transl Med* 2017; 5:310. 28856150 10.21037/atm.2017.06.14 PMC5555990

[2] Gottdiener JS. The shape of LVH in hypertension. *JACC Cardiovasc Imaging* 2015; 8:1042–1044. 26381766 10.1016/j.jcmg.2015.08.002

[3] Williams B Mancia G Spiering W Agabiti Rosei E Azizi M Burnier M. 2018 ESC/ESH Guidelines for the management of arterial hypertension: The Task Force for the management of arterial hypertension of the European Society of Cardiology and the European Society of Hypertension: The Task Force for the management of arterial hypertension of the European Society of Cardiology and the European Society of Hypertension. *J Hypertens* 2018; 36:1953–2041. 30234752 10.1097/HJH.0000000000001940

[4] Mancia G Kreutz R Brunström M Burnier M Grassi G Januszewicz A. 2023 ESH Guidelines for the management of arterial hypertension The Task Force for the management of arterial hypertension of the European Society of Hypertension: Endorsed by the International Society of Hypertension (ISH) and the European Renal Association (ERA). *J Hypertens* 2023; 41:1874–2071. 37345492 10.1097/HJH.0000000000003480

[5] Rodrigues JCL Amadu AM Dastidar AG Szantho GV Lyen SM Godsave C. Comprehensive characterisation of hypertensive heart disease left ventricular phenotypes. *Heart* 2016; 102:1671–1679. 27260191 10.1136/heartjnl-2016-309576 PMC5099214

[6] Ha ET Ivanov A Yeboah J Seals A Peterson SJ Parikh M. Relation of left ventricular hypertrophy subtype to long-term mortality in those with subclinical cardiovascular disease (from the multiethnic study of atherosclerosis [MESA]). *Am J Cardiol* 2022; 175:131–138. 35550820 10.1016/j.amjcard.2022.03.058

[7] Prognosis of left ventricular geometric patterns in the Framingham heart study. *J Am Coll Cardiol* 1995; 25:879–884. 7884091 10.1016/0735-1097(94)00473-4

[8] Schillaci G Verdecchia P Borgioni C Ciucci A Guerrieri M Zampi I. Improved electrocardiographic diagnosis of left ventricular hypertrophy. *Am J Cardiol* 1994; 74:714–719. 7942532 10.1016/0002-9149(94)90316-6

[9] Bacharova L Estes EH Jr Hill JA Pahlm O Schillaci G Strauss D. Changing role of ECG in the evaluation left ventricular hypertrophy. *J Electrocardiol* 2012; 45:609–611. 23022306 10.1016/j.jelectrocard.2012.08.010

[10] Schillaci G Battista F Pucci G. A review of the role of electrocardiography in the diagnosis of left ventricular hypertrophy in hypertension. *J Electrocardiol* 2012; 45:617–623. 23022303 10.1016/j.jelectrocard.2012.08.051

[11] Whelton PK Carey RM Aronow WS Donald E Casey J Collins KJ. 2017 ACC/AHA/AAPA/ABC/ACPM/AGS/APhA/ASH/ASPC/NMA/PCNA guideline for the prevention, detection, evaluation, and management of high blood pressure in adults: a report of the American College of Cardiology/American Heart Association Task Force on Clinical Practice Guidelines. *Hypertension* 2018; 71:1269–1324. 29133354 10.1161/HYP.0000000000000066

[12] Minardi J D’Angelo J Davis S Davidov D. 100 are emergency physicians good enough at detecting left ventricular hypertrophy on electrocardiogram? *Ann Emerg Med* 2012; 60:S37.

[13] Sahota G Singh J. Interpretation of electrocardiograms in primary care. *Br J Gen Pract* 2016; 66:406. 10.3399/bjgp16X686293 PMC4979934 27481967

[14] Begg G Willan K Tyndall K Pepper C Tayebjee M. Electrocardiogram interpretation and arrhythmia management: a primary and secondary care survey. *Br J Gen Pract* 2016; 66:e291–e296. 27025557 10.3399/bjgp16X684781 PMC4838440

[15] Naderi H Ramírez J van Duijvenboden S Pujadas ER Aung N Wang L. Predicting left ventricular hypertrophy from the 12-lead electrocardiogram in the UK Biobank imaging study using machine learning. *Eur Heart J Digit Health* 2023; 4:316–324. 37538142 10.1093/ehjdh/ztad037 PMC10393938

[16] Petersen SE Matthews PM Bamberg F Bluemke DA Francis JM Friedrich MG. Imaging in population science: cardiovascular magnetic resonance in 100,000 participants of UK Biobank - rationale, challenges and approaches. *J Cardiovasc Magn Reson* 2013; 15:46. 23714095 10.1186/1532-429X-15-46 PMC3668194

[17] Petersen SE Matthews PM Francis JM Robson MD Zemrak F Boubertakh R. UK Biobank's cardiovascular magnetic resonance protocol. *J Cardiovasc Magn Reson* 2016; 18:8. 26830817 10.1186/s12968-016-0227-4 PMC4736703

[18] Bai W Sinclair M Tarroni G Oktay O Rajchl M Vaillant G. Automated cardiovascular magnetic resonance image analysis with fully convolutional networks. *J Cardiovasc Magn Reson* 2018; 20:65. 30217194 10.1186/s12968-018-0471-x PMC6138894

[19] Tobin MD Sheehan NA Scurrah KJ Burton PR. Adjusting for treatment effects in studies of quantitative traits: antihypertensive therapy and systolic blood pressure. *Stat Med* 2005; 24:2911–2935. 16152135 10.1002/sim.2165

[20] Cui JS Hopper JL Harrap SB. Antihypertensive treatments obscure familial contributions to blood pressure variation. *Hypertension* 2003; 47:207–210. 10.1161/01.hyp.0000044938.94050.e3 12574083

[21] de Marvao A McGurk KA Zheng SL Thanaj M Bai W Duan J. Phenotypic expression and outcomes in individuals with rare genetic variants of hypertrophic cardiomyopathy. *J Am Coll Cardiol* 2021; 78:1097–1110. 34503678 10.1016/j.jacc.2021.07.017 PMC8434420

[22] Mosteller RD. Simplified calculation of body-surface area. *New Engl J Med* 1987; 317:1098. 3657876 10.1056/NEJM198710223171717

[23] MATLAB - MathWorks. Available at: https://uk.mathworks.com/products/matlab.html. (Accessed 23 August 2021)

[24] Nissen SE, Tuzcu EM, Schoenhagen P, Crowe T, Sasiela WJ, Tsai J, *et al.* Statin therapy, LDL cholesterol, c-reactive protein, and coronary artery disease. Available at: 10.1056/NEJMoa042000. 2005. doi:10.1056/NEJMoa042000. 15635110

[25] Fit multiclass models for support vector machines or other classifiers - MATLAB fitcecoc - MathWorks United Kingdom. Available at: https://uk.mathworks.com/help/stats/fitcecoc.html. (Accessed 28 July 2022)

[26] Fit ensemble of learners for classification - MATLAB fitcensemble - MathWorks United Kingdom. Available at: https://uk.mathworks.com/help/stats/fitcensemble.html. (Accessed 5 December 2022)

[27] Ruopp MD Perkins NJ Whitcomb BW Schisterman EF. Youden index and optimal cut-point estimated from observations affected by a lower limit of detection. *Biometr J* 2008; 50:419. 10.1002/bimj.200710415 PMC2515362 18435502

[28] Völzke H. Study of Health in Pomerania (SHIP). *Bundesgesundheitsblatt Gesundheitsforschung Gesundheitsschutz* 2012; 55:790–794. [in German]. 22736157 10.1007/s00103-012-1483-6

[29] Völzke H Schössow J Schmidt CO Jürgens C Richter A Werner A. Cohort profile update: the Study of Health in Pomerania (SHIP). *Int J Epidemiol* 2022; 51:e372–e383. 35348705 10.1093/ije/dyac034

[30] Bülow R Ittermann T Dörr M Poesch A Langner S Völzke H. Reference ranges of left ventricular structure and function assessed by contrast-enhanced cardiac MR and changes related to ageing and hypertension in a population-based study. *Eur Radiol* 2018; 28:3996–4005. 29541910 10.1007/s00330-018-5345-y

[31] Bycroft C Freeman C Petkova D Band G Elliott LT Sharp K. The UK Biobank resource with deep phenotyping and genomic data. *Nature* 2018; 562:203–209. 30305743 10.1038/s41586-018-0579-z PMC6786975

[32] RStudio | Open source & professional software for data science teams. Available at: https://rstudio.com/. (Accessed 23 August 2021).

[33] Kuhn M. *The caret Package*. Available at: https://topepo.github.io/caret/. (Accessed 27 July 2022).

[34] van Buuren S Groothuis-Oudshoorn K. mice: multivariate imputation by chained equations in R. *J Stat Soft* 2011; 45:1–67.

[35] Jakobsen JC Gluud C Wetterslev J Winkel P. When and how should multiple imputation be used for handling missing data in randomised clinical trials - a practical guide with flowcharts. *BMC Med Res Methodol* 2017; 17:1–10. 29207961 10.1186/s12874-017-0442-1 PMC5717805

[36] Dong Y Peng C-YJ. Principled missing data methods for researchers. *SpringerPlus* 2013; 2:1–17. 23853744 10.1186/2193-1801-2-222 PMC3701793

[37] Bang CN Gerdts E Aurigemma GP Boman K Simone G de Dahlöf B. Four-group classification of left ventricular hypertrophy based on ventricular concentricity and dilatation identifies a low-risk subset of eccentric hypertrophy in hypertensive patients. *Circ Cardiovasc Imaging* 2014; 7:422–429. 24723582 10.1161/CIRCIMAGING.113.001275

[38] Nauta JF Hummel YM Tromp J Ouwerkerk W Meer P van der Jin X. Concentric vs. eccentric remodelling in heart failure with reduced ejection fraction: clinical characteristics, pathophysiology and response to treatment. *Eur J Heart Fail* 2020; 22:1147. 31713324 10.1002/ejhf.1632 PMC7540540

[39] Bacharova L. Electrical and structural remodeling in left ventricular hypertrophy—a substrate for a decrease in QRS voltage? *Ann Noninvasive Electrocardiol* 2007; 12:260–273. 17617072 10.1111/j.1542-474X.2007.00170.x PMC6932385

[40] Levy D Labib SB Anderson KM Christiansen JC Kannel WB Castelli WP. Determinants of sensitivity and specificity of electrocardiographic criteria for left ventricular hypertrophy. *Circulation* 1990; 81:815–820. 2137733 10.1161/01.cir.81.3.815

[41] Tison GH Zhang J Delling FN Deo RC. Automated and Interpretable Patient ECG Profiles for Disease Detection, Tracking, and Discovery. *Circ Cardiovasc Qual Outcomes* 2019; 12:e005289. 31525078 10.1161/CIRCOUTCOMES.118.005289 PMC6951431

[42] Kwon J-M Jeon K-H Kim HM Kim MJ Lim SM Kim K-H. Comparing the performance of artificial intelligence and conventional diagnosis criteria for detecting left ventricular hypertrophy using electrocardiography. *Europace* 2020; 22:412–419. 31800031 10.1093/europace/euz324

[43] Khurshid S Friedman S Pirruccello JP Di Achille P Diamant N Anderson CD. Deep learning to predict cardiac magnetic resonance-derived left ventricular mass and hypertrophy from 12-lead ECGs. *Circ Cardiovasc Imaging* 2021; 14:e012281. 34126762 10.1161/CIRCIMAGING.120.012281 PMC8217289

[44] Beneyto M Ghyaza G Cariou E Amar J Lairez O. Development and validation of machine learning algorithms to predict left ventricular hypertrophy etiology. *Arch Cardiovasc Dis Suppl* 2023; 15:109. 10.1016/j.acvd.2023.06.005 37474391

[45] Alfakih K Bloomer T Bainbridge S Bainbridge G Ridgway J Williams G. A comparison of left ventricular mass between two-dimensional echocardiography, using fundamental and tissue harmonic imaging, and cardiac MRI in patients with hypertension. *Eur J Radiol* 2004; 52:103–109. 15489067 10.1016/j.ejrad.2003.09.015

[46] Foppa M Duncan BB Rohde LE. Echocardiography-based left ventricular mass estimation. How should we define hypertrophy? *Cardiovasc Ultrasound* 2005; 3:1–13. 15963236 10.1186/1476-7120-3-17 PMC1183230

[47] Myerson SG Bellenger NG Pennell DJ. Assessment of left ventricular mass by cardiovascular magnetic resonance. *Hypertension* 2002; 39:750–755. 11897757 10.1161/hy0302.104674

[48] Bluemke DA Kronmal RA Lima JAC Liu K Olson J Burke GL. The relationship of left ventricular mass and geometry to incident cardiovascular events. *J Am Coll Cardiol* 2008; 52:2148–2155. 19095132 10.1016/j.jacc.2008.09.014 PMC2706368

[49] Petersen SE Aung N Sanghvi MM Zemrak F Fung K Paiva JM. Reference ranges for cardiac structure and function using cardiovascular magnetic resonance (CMR) in Caucasians from the UK Biobank population cohort. *J Cardiovasc Magn Reson* 2017; 19:18. 28178995 10.1186/s12968-017-0327-9 PMC5304550

[50] Slivnick J Lampert BC. Hypertension and heart failure. *Heart Fail Clin* 2019; 15:531–541. 31472888 10.1016/j.hfc.2019.06.007

[51] Messerli FH Rimoldi SF Bangalore S. The transition from hypertension to heart failure: contemporary update. *JACC Heart Fail* 2017; 5:543–551. 28711447 10.1016/j.jchf.2017.04.012

[52] Recommendations | Hypertension in adults: diagnosis and management | Guidance | NICE. Available at: https://www.nice.org.uk/guidance/ng136/chapter/Recommendations. (Accessed 18 July 2022).

[53] Chen T, Guestrin C. Xgboost: a scalable tree-boosting system. In proceedings of the 22nd ACM sigkdd international conference on knowledge discovery and data mining. 2016: 785–794.

[54] Blockeel H Devos L Frénay B Nanfack G Nijssen S. Decision trees: from efficient prediction to responsible AI. *Frontiers in Artificial Intelligence* 2023; 6:1124553. 37565044 10.3389/frai.2023.1124553 PMC10411911

[55] Cunningham P Delaney SJ. K-Nearest neighbor classifiers – a tutorial. *ACM computing surveys* 2021; 6:1–25.

