# Supplementary material for: Diagnostic and prognostic value of ECG-predicted hypertension-mediated left ventricular hypertrophy using machine learning
Source: J Hypertens. 2025 May 23;43(8):1327–38. doi: 10.1097/HJH.0000000000004034 (PMC12237117; doi:10.1097/HJH.0000000000004034)
Supplement: Supplemental Digital Content [file jhype-43-1327-s001.docx]

**Supplementary material**

**Supplementary Table 1.** UK Biobank data fields used to identify hypertensive participants.

| **Phenotype** | **Data fields** | **Field names** | **Data code definitions** |
| --- | --- | --- | --- |
| Hypertension | 20002 | Non-cancer illness code, self-reported | Hypertension, Essential hypertension |
| Hypertension | 6150 | Vascular/heart problems diagnosed by doctor | High blood pressure |
| Hypertension | 6177 | Medication for cholesterol, blood pressure or diabetes | Blood pressure medication |
| Hypertension | 6153 | Medication for cholesterol, blood pressure, diabetes or take exogenous hormones | Blood pressure medication |
| Hypertension | 4080 | Systolic blood pressure, automated reading | ≥130 mmHg |
| Hypertension | 4079 | Diastolic blood pressure, automated reading | ≥85 mmHg |
| Hypertension | 93 | Systolic blood pressure, manual reading | ≥130 mmHg |
| Hypertension | 94 | Diastolic blood pressure, manual reading | ≥85 mmHg |
| Aortic stenosis | 20002 | Non-cancer illness code, self-reported | Aortic stenosis |
| Hypertrophic cardiomyopathy | 20002 | Non-cancer illness code, self-reported | Hypertrophic cardiomyopathy |

**Supplementary Table 2.** Definition of ECG biomarkers associated with LVH.

| **ECG marker** | **Definition** |
| --- | --- |
| Sokolow-Lyon index | (SV1 or SV2) + (RV5 or RV6) >35mm or R wave in aVL >=11mm |
| Cornell voltage | SV3 +RaVL >28mm (men) >20mm (women) |
| Pathological Q waves | >30ms in duration and >1/3 of the R wave in depth in two or more contiguous leads (I, II, V1-6) |
| R wave amplitude (mV) | Amplitude of R wave in V5 or V6 >2.6mV, in aVL>1.1mV |
| QRS amplitude (mV) | Absolute value of the difference between maximum and minimum of the QRS complex values |
| QRS duration (ms) | QRS width from beginning of Q wave to end of S wave, (>=90ms) |
| QRS ascending slope | Upward slope of QRS complex |
| QRS descending slope | Downward slope of QRS complex |
| QTc interval (ms) | Prolonged corrected QT >=450ms (men) >=460ms (women) |
| STT segment amplitude | Absolute value of the difference between maximum and minimum of the ST segment values |
| ST segment displacement | Difference between the beginning of the QRS complex and the beginning of the T wave |
| QTc dispersion | Inter lead variations in QT segment length (<40ms) |
| T wave inversion | T wave amplitude ≤ −0.1mV or a negative inflexion of at least 0.1mV in lead I, II or V3-6 |
| T wave amplitude (mV) | Absolute value of the difference between maximum and minimum of the T wave complex values |
| T wave axis (∘) | <-15º to ≥-180º or >105º to ≤180º |
| T peak to T end interval (ms) | Duration between the peak of the T wave and the end of the T wave in each lead |

**Supplementary Table 3.** Codes used to define clinical outcomes using ICD-9, ICD-10and OPCS4 codes.

| **Myocardial Infarction** | |
| --- | --- |
| **ICD10 codes** | **Definition** |
| I21 | Acute myocardial infarction |
| I21.0 | Acute transmural myocardial infarction of anterior wall |
| I21.1 | Acute transmural myocardial infarction of inferior wall |
| I21.2 | Acute transmural myocardial infarction of other sites |
| I21.3 | Acute transmural myocardial infarction of unspecified site |
| I21.4 | Acute subendocardial myocardial infarction |
| I21.9 | Acute myocardial infarction, unspecified |
| I22 | Subsequent myocardial infarction |
| I22.0 | Subsequent myocardial infarction of anterior wall |
| I22.1 | Subsequent myocardial infarction of inferior wall |
| I22.8 | Subsequent myocardial infarction of other sites |
| I22.9 | Subsequent myocardial infarction of unspecified site |
| I23 | Certain current complications following acute myocardial infarction |
| I23.0 | Haemopericardium as current complication following acute myocardial infarction |
| I23.1 | Atrial septal defect as current complication following acute myocardial infarction |
| I23.2 | Ventricular septal defect as current complication following acute myocardial infarction |
| I23.3 | Rupture of cardiac wall without haemoprericardium as current complication following acute myocardial infarction |
| I23.5 | Rupture of papillary muscle as current complication following acute myocardial infarction |
| I23.6 | Thrombosis of atrium, auricular appendage and ventricle as current complications following acute myocardial infarction |
| I23.8 | Other current complications following acute myocardial infarction |
| I24 | Other acute ischaemic heart disease |
| I24.0 | Coronary thrombosis not resulting in myocardial infarction |
| I24.1 | Dressler’s syndrome |
| I24.8 | Other forms of acute ischaemic heart disease |
| I24.9 | Acute ischaemic heart disease, unspecified |
| I25 | Chronic ischaemic heart disease |
| I25.0 | Atherosclerotic cardiovascular disease |
| I25.1 | Atherosclerotic heart disease |
| I25.2 | Old myocardial infarction |
| I25.3 | Aneurysm of heart |
| I25.4 | Coronary artery aneurysm |
| I25.5 | Ischaemic cardiomyopathy |
| I25.6 | Silent myocardial ischaemia |
| I25.8 | Other forms of chronic ischaemic heart disease |
| I25.9 | Chronic ischaemic heart disease, unspecified |
| **ICD9 codes** | **Definition** |
| 4109 | Acute myocardial infarction |
| **OPCS4** | **Definition** |
| K40 | Saphenous vein graft replacement of coronary artery |
| K40.1 | Saphenous vein graft replacement of one coronary artery |
| K40.2 | Saphenous vein graft replacement of two coronary arteries |
| K40.3 | Saphenous vein graft replacement of three coronary arteries |
| K40.4 | Saphenous vein graft replacement of four or more coronary arteries |
| K40.8 | Other specified saphenous vein graft replacement of coronary artery |
| K40.9 | Unspecified saphenous vein graft replacement of coronary artery |
| K41 | Other autograft replacement of coronary artery |
| K41.1 | Autograft replacement of one coronary artery NEC |
| K41.2 | Autograft replacement of two coronary arteries NEC |
| K41.3 | Autograft replacement of three coronary arteries NEC |
| K41.4 | Autograft replacement of four or more coronary arteries NEC |
| K41.8 | Other specified other autograft replacement of coronary artery |
| K41.9 | Unspecified other autograft replacement of coronary artery |
| K42 | Allograft replacement of coronary artery |
| K42.1 | Allograft replacement of one coronary artery |
| K42.2 | Allograft replacement of two coronary arteries |
| K42.3 | Allograft replacement of three coronary arteries |
| K42.4 | Allograft replacement of four or more coronary arteries |
| K42.8 | Other specified other allograft replacement of coronary artery |
| K42.9 | Unspecified other allograft replacement of coronary artery |
| K44 | Other replacement of coronary artery |
| K44.1 | Replacement of coronary arteries using multiple methods |
| K44.2 | Revision of replacement of coronary artery |
| K44.8 | Other specified other replacement of coronary artery |
| K44.9 | Unspecified other replacement of coronary artery |
| K45 | Connection of thoracic artery to coronary artery |
| K45.1 | Double anastomosis of mammary arteries to coronary arteries |
| K45.2 | Double anastomosis of thoracic arteries to coronary arteries NEC |
| K45.3 | Anastomosis of mammary artery to left anterior descending coronary artery |
| K45.4 | Anastomosis of mammary artery to coronary artery NEC |
| K45.5 | Anastomosis of thoracic artery to coronary artery NEC |
| K45.6 | Revision of connection of thoracic artery to coronary artery |
| K45.8 | Other specified connection of thoracic artery to coronary artery |
| K45.9 | Unspecified connection of thoracic artery to coronary aartery |
| K49 | Transluminal balloon angioplasty of coronary artery |
| K49.1 | Percutaneous transluminal balloon angioplasty of one coronary artery |
| K49.2 | Percutaneous transluminal balloon angioplasty of multiple coronary arteries |
| K49.3 | Percutaneous transluminal balloon angioplasty of bypass graft of coronary artery |
| K49.4 | Percutaneous transluminal cutting balloon angioplasty of coronary artery |
| K49.8 | Other specified transluminal balloon angioplasty of coronary artery |
| K49.9 | Unspecified transluminal balloon angioplasty of coronary artery |
| K50 | Other therapeutic transluminal operations on coronary artery |
| K50.1 | Percutaneous transluminal laser coronary angioplasty |
| K50.2 | Percutaneous transluminal coronary thrombolysis using streptokinase |
| K50.3 | Percutaneous transluminal injection of therapeutic substance into coronary artery NEC |
| K50.4 | Percutaneous transluminal atherectomy of coronary artery |
| K50.8 | Other specified other therapeutic transluminal; operations on coronary artery |
| K50.9 | Unspecified other therapeutic transluminal operations on coronary artery |
| K75 | Percutaneous transluminal balloon angioplasty and insertion of stent into coronary artery |
| K75.1 | Percutaneous transluminal balloon angioplasty and insertion of 1-2 drug-eluting stents into coronary artery |
| K75.2 | Percutaneous transluminal balloon angioplasty and insertion of 3 or more drug-eluting stents into coronary artery |
| K75.3 | Percutaneous transluminal balloon angioplasty and insertion of 1-2 stents into coronary artery |
| K75.4 | Percutaneous transluminal balloon angioplasty and insertion of 3 or more stents into coronary artery NEC |
| K75.8 | Other specified percutaneous transluminal balloon angioplasty and insertion of stent into coronary artery |
| K75.9 | Unspecified percutaneous transluminal balloon angioplasty and insertion of stent into coronary artery |
| **Heart Failure** | |
| **ICD10 codes** | **Definition** |
| I11.0 | Hypertensive heart disease with (congestive) heart failure |
| I13.0 | Hypertensive heart and renal disease with (congestive) heart failure |
| I13.2 | Hypertensive heart and renal disease with both (congestive) heart failure and renal failure |
| I25.5 | Ischaemic cardiomyopathy |
| I50 | Heart failure |
| I50.0 | Congestive heart failure |
| I50.1 | Left ventricular failure |
| I50.9 | Heart failure, unspecified |
| J81 | Pulmonary oedema |
| K76.1 | Chronic passive congestion of liver |
| **ICD9 codes** | **Definition** |
| 4280 | Congestive heart failure |
| 4281 | Left heart failure |
| 4289 | Heart failure, unspecified |
| **OPCS4 codes** | **Definition** |
| K59.6 | Implantation of cardioverter defibrillator using three electrode leads |
| K61.7 | Implantation of biventricular cardiac pacemaker system |
| K60.7 | Implantation of intravenous biventricular cardiac pacemaker system |
| **Stroke** | |
| **ICD10 codes** | **Definition** |
| I60 | Subarachnoid haemorrhage |
| I60.0 | Subarachnoid haemorrhage from carotid siphon bifurcation |
| I60.1 | Subarachnoid haemorrhage from middle cerebral artery |
| I60.2 | Subarachnoid haemorrhage from anterior communicating artery |
| I60.3 | Subarachnoid haemorrhage from posterior communicating artery |
| I60.4 | Subarachnoid haemorrhage from basilar artery |
| I60.5 | Subarachnoid haemorrhage from vertebral artery |
| I60.6 | Subarachnoid haemorrhage from other intracranial arteries |
| I60.7 | Subarachnoid haemorrhage from intracranial artery, unspecified |
| I60.8 | Other subarachnoid haemorrhage |
| I60.9 | Subarachnoid haemorrhage, unspecified |
| I61 | Intracerebral haemorrhage |
| I61.0 | Intracerebral haemorrhage in hemisphere subcortical |
| I61.1 | Intracerebral haemorrhage in hemisphere, cortical |
| I61.2 | Intracerebral haemorrhage in hemisphere, unspecified |
| I61.3 | Intracerebral haemorrhage in brain stem |
| I61.4 | Intracerebral haemorrhage in cerebellum |
| I61.5 | Intracerebral haemorrhage, intraventricular |
| I61.6 | Intracerebral haemorrhage, multiple localised |
| I61.8 | Other intracerebral haemorrhage |
| I61.9 | Intracerebral haemorrhage, unspecified |
| I62 | Other nontraumatic intracranial haemorrhage |
| I62.0 | Subdural haemorrhage (acute) (nontraumatic) |
| I62.1 | Nontraumatic extradural haemorrhage |
| I62.9 | Intracranial haemorrhage (nontraumatic), unspecified |
| I63 | Cerebral infarction |
| I63.0 | Cerebral infarction due to thrombosis of precerebral arteries |
| I63.1 | Cerebral infarction due to embolism of precerebral arteries |
| I63.2 | Cerebral infarction due to unspecified occlusion or stenosis of precerebral arteries |
| I63.3 | Cerebral infarction due to thrombosis of cerebral arteries |
| I63.4 | Cerebral infarction due to embolism of cerebral arteries |
| I63.5 | Cerebral infarction due to unspecified occlusion or stenosis of cerebral arteries |
| I63.6 | Cerebral infarction due to cerebral venous thrombosis, nonpyogenic |
| I63.8 | Other cerebral infarction |
| I63.9 | Cerebral infarction, unspecified |
| I64 | Stroke, not specified as haemorrhage or infarction |
| I65 | Occlusion and stenosis of precerebral arteries, not resulting in cerebral infarction |
| I65.0 | Occlusion and stenosis of vertebral artery |
| I65.1 | Occlusion and stenosis of basilar artery |
| I65.2 | Occlusion and stenosis of carotid artery |
| I65.3 | Occlusion and stenosis of multiple and bilateral precerebral arteries |
| I65.8 | Occlusion and stenosis of other precerebral artery |
| I65.9 | Occlusion and stenosis of unspecified precerebral artery |
| I66 | Occlusion and stenosis of cerebral arteries, not resulting in cerebral infarction |
| I66.0 | Occlusion and stenosis of middle cerebral artery |
| I66.1 | Occlusion and stenosis of anterior cerebral artery |
| I66.2 | Occlusion and stenosis of posterior cerebral artery |
| I66.3 | Occlusion and stenosis of cerebellar arteries |
| I66.4 | Occlusion and stenosis of multiple and bilateral cerebral arteries |
| I66.8 | Occlusion and stenosis of other cerebral artery |
| I66.9 | Occlusion and stenosis of unspecified cerebral artery |
| I67.0 | Dissection of cerebral arteries, nonruptured |
| I67.8 | Other specified cerebrovascular diseases |
| I67.9 | Cerebrovascular disease, unspecified |
| I69 | Sequelae of cerebrovascular disease |
| **ICD9 codes** | **Definition** |
| 4309 | Subarachnoid haemorrhage |
| 4319 | Intracerebral haemorrhage |
| 4320 | Nontraumatic extradural haemorrhage |
| 4321 | Subdural haemorrhage |
| 4331 | Occlusion and stenosis of carotid artery |
| 4339 | Occlusion and stenosis of precerebral arteries, unspecified |
| 4349 | Occlusion of cerebral arteries, unspecified |
| 4369 | Acute but ill-defined cerebrovascular disease |
| 4371 | Other generalised ischaemic cerebrovascular disease |
| **OPCS4 codes** | **Definition** |
| L35.4 | Percutaneous transluminal embolectomy of cerebral artery |
| **Ventricular Arrythmias** | |
| **ICD10 codes** | **Definition** |
| I47.2 | Ventricular tachycardia |
| I49.0 | Ventricular fibrillation and flutter |
| I46.0 | Cardiac arrest with successful resuscitation |
| I46.1 | Sudden cardiac death, so described |
| I46.9 | Cardiac arrest, unspecified |
| I47.0 | Re-entry ventricular arrythmia |
| **ICD9 codes** | **Definition** |
| 4270 | Paroxysmal ventricular tachycardia |
| 4271 | Paroxysmal ventricular tachycardia |
| 4272 | Paroxysmal tachycardia, unspecified |
| 4274 | Ventricular fibrillation and flutter |
| **OPCS4 codes** | **Definition** |
| K59 | Cardioverter defibrillator introduced through vein |
| K59.1 | Implantation of cardioverter defibrillator using one electrode lead |
| K59.2 | Implantation of cardioverter defibrillator using two electrode leads |
| K59.3 | Resitting of leads of cardioverter defibrillator |
| K59.4 | Renewal of cardioverter defibrillator |
| K59.6 | Implantation of cardioverter defibrillator using three electrode leads |
| K59.8 | Other specified cardioverter defibrillator introduced through the vein |
| K59.9 | Unspecified cardioverter defibrillator introduced through the vein |
| K72 | Other cardioverter defibrillator |
| K72.1 | Implantation of subcutaneous cardioverter defibrillator |
| K72.3 | Renewal of subcutaneous cardioverter defibrillator |

**Supplementary Table 4. Associations of ECG-predicted hypertension-mediated LVH phenotypes and clinical outcomes.**

|  | **MACE** | | **Heart Failure** | |
| --- | --- | --- | --- | --- |
|  | HR (95% CI) | P-value | HR (95% CI) | P-value |
| **LV remodeling** | 1.40 (0.37-2.76) | 0.1 | 1.55 (071.-3.36) | 0.3 |
| **Eccentric LVH** | 0.93 (0.42-2.04) | 0.9 | 3.24 (1.06-9.86) | 0.04 |
| **Concentric LVH** | 1.01 (0.37-2.76) | 0.1 | 1.27 (0.17-9.65) | 0.8 |

*AUC: area under the receiver operator curve; CI: confidence interval; HR: hazard ratio; MACE: major adverse cardiovascular events; LVH: left ventricular hypertrophy.*
